# The association between body composition and orthostatic hypotension in patients with neurodegenerative disorders in parkinsonism-related multidisciplinary clinic

**DOI:** 10.3389/fnagi.2026.1830578

**Published:** 2026-07-07

**Authors:** Jin Fu, Rui Wang, Wengkei Wong, Yichun Wang, Yuanyuan Bao, Han Wang, Yanping Liu, Kang Yu

**Affiliations:** 1 Department of Clinical Nutrition, Peking Union Medical College Hospital, Chinese Academy of Medical Sciences and Peking Union Medical College, Beijing, China; 2 Department of Clinical Nutrition, Shanghai Ninth People’s Hospital, Shanghai, China; 3 Department of Clinical Nutrition, Peking Union Medical College Hospital Macau Medical Center, Avenida do Hospital do Cotai, Macau, China; 4 Department of Neurology, Peking Union Medical College Hospital, Chinese Academy of Medical Sciences and Peking Union Medical College, Beijing, China

**Keywords:** body composition, muscle mass, neurodegeneration, orthostatic hypotension, sarcopenia

## Abstract

**Introduction:**

Orthostatic hypotension (OH) commonly impairs the quality of life and prognosis of patients with neurodegenerative disorders. Since skeletal muscle functions as a peripheral pump for blood pressure stabilization and a reservoir for intracellular water, we investigated its association with body composition in these patients.

**Methods:**

This prospective cross-sectional study included 86 patients with neurodegenerative disorders from Parkinsonism-related Multidisciplinary Clinic in Peking Union Medical College Hospital (PUMCH), including multiple system atrophy (MSA), progressive supranuclear palsy (PSP) and other atypical parkinsonian syndromes (APS). OH was defined as a sustained reduction in systolic blood pressure of ≥20 mmHg or diastolic blood pressure of ≥10 mmHg within 3 min of active standing. Participants were divided into with OH and without OH (non-OH). Body composition was measured via Bioelectrical Impedance Analysis (BIA). The measured values were normalized to height squared to generating indices such as the fat-free mass index (FFMI) and appendicular muscle mass index (AMI). Clinical scores, levodopa equivalent daily dosage (LEDD), and grip strength were compared between groups. Sarcopenia was diagnosed according to the Asian Working Group for Sarcopenia 2019 (AWGS2019) criteria.

**Results:**

The incidence of OH was 45.3%, significantly higher in MSA (58.0%) than in PSP (13.3%). Sarcopenia prevalence did not differ significantly between groups. Elastic net and support vector machine (SVM) analysis identified lower limb extracellular water index (LLEWI) and AMI as the most predictive body composition indicators for OH. Linear regression confirmed AMI as an independent negative predictor of orthostatic systolic blood pressure change (*β* = −4.69, *p* = 0.008).

**Conclusion:**

Our findings indicate that OH is significantly associated with decreased AMI and lower LLEWI. Measuring these indices may offer valuable insights into OH risk, potentially serving as a more sensitive early indicator than a formal diagnosis of sarcopenia.

## 1 Introduction

Orthostatic hypotension (OH) is a clinical condition characterized by a significant drop in blood pressure upon standing from a supine position. It is typically defined as a sustained reduction in systolic blood pressure of ≥20 mmHg or a decrease in diastolic blood pressure of ≥10 mmHg within 3 min after posture change, such as during active standing or a head-up tilt test (Freeman et al., 2011). While OH may present with positional symptoms such as dizziness and syncope, it can also be asymptomatic (subclinical), which further explains its frequent underdiagnosis. Typically, OH is classified as non-neurogenic (non-NOH), often due to medications or volume loss, or neurogenic (NOH), resulting from impaired cardiovascular autonomic regulation. A heart rate variability-to-systolic blood pressure variability ratio ( Δ HR/ Δ SBP) below 0.5 bpm/mmHg suggests NOH. However, these categories are not mutually exclusive and may coexist. Prior studies indicate that OH is commonly associated with neurodegenerative diseases: its prevalence ranges from 37% to 58% in Parkinson’s disease (PD) (Allcock et al., 2004), increasing with age and disease duration; from 30% to 50% with Lewy bodies (DLBs) (Thaisetthawatkul et al., 2004); and up to 75% in multiple system atrophy (MSA) (Xie et al., 2022). Many patients experience early-onset and obvious symptoms which substantially impair quality of life (Wieling et al., 2022), contribute to falls (Wood et al., 2002) and cognitive decline, and are associated with reduced survival (Benvenuto and Krakoff, 2011).

Treatment of OH primarily involves lifestyle management and pharmacotherapy. Due to the limited options and efficacy of medication, both domestic and international OH guidelines emphasize initiating non-pharmacologic interventions —such as adequate hydration, salt intake, and correction of dehydration —as early as possible. Although theoretically effective, poor adherence often limits the success of lifestyle measures, leaving OH treatment an unmet clinical need. Previous studies indicate that severe sarcopenia is associated with a higher likelihood of OH or greater supine-to-standing blood pressure changes (Duggan et al., 2023b; Soysal et al., 2020; Keskin et al., 2021). Moreover, upper arm circumference, calf circumference, and hand grip strength have been identified as potential risk factors for OH in older hypertensive patients (Zhang et al., 2022). The underlying mechanisms may involve muscle pumping during standing, which enhances venous return from the lower extremities (Hellebrandt and Franseen, 1943) and the role of muscles as the major reservoir of intracellular water, helping to buffer fluctuations of circulation volume (Benton and Schlairet, 2016). Therefore, we hypothesize that assessing muscle body composition could earlier reveal the body’s buffering capacity against OH, thereby improving OH prediction and enabling more individualized and refined treatment guidance.

This study aimed to, (1) investigate the prevalence of OH among non-Parkinson’s disease patients attending a Parkinsonism-related multidisciplinary team (PD-MDT) clinic; (2) prospectively observe the correlation between OH and body composition, especially muscle related parameters, in patients with neurodegenerative diseases.

## 2 Materials and methods

### 2.1 Subject and groups

A cross-sectional observational study was conducted, and data from 114 patients with PD and movement disorders who underwent multidisciplinary (MDT) consultation at Peking Union Medical College Hospital (PUMCH) from October 2022 to April 2024 were prospectively and continuously collected. The inclusion criteria were as follows: patients with a clinical diagnosis of neurological degeneration according to the corresponding diagnostic criteria, which included multiple system atrophy (MSA; diagnosed according to the 2022 Movement Disorder Society [MDS] criteria) (Wenning et al., 2022), progressive supranuclear palsy (PSP; diagnosed according to the 2017 MDS criteria) (Höglinger et al., 2017), and other atypical Parkinsonian syndromes (APSs). Specifically, the APS category encompassed conditions such as Dementia with Lewy Bodies (DLB; diagnosed via the 2017 McKeith consensus criteria) (McKeith et al., 2017), as well as undifferentiated atypical parkinsonism. Patients classified as having undifferentiated atypical parkinsonism exhibited general features of parkinsonism and presented with atypical ‘red flags’ that clinically excluded idiopathic Parkinson’s disease (based on the 2015 MDS clinical diagnostic criteria) (Postuma et al., 2015), yet they did not fully satisfy the definitive consensus criteria for MSA or PSP at the baseline evaluation. Given the cross-sectional design of this study, participants were grouped strictly according to their clinical diagnostic status at the time of enrolment. MSA was divided into Parkinson’s type (MSA-p) and cerebellar type (MSA-c) according to the main clinical manifestations. The exclusion criteria were: (1) severe functional impairment precluding the ability to stand or complete body composition assessment and lying-standing BP recording; (2) chronic use of alpha-blockers, diuretics, or other OH-inducing medications (levodopa drugs were considered separately); (3) a history of conditions associated with non-neurodegenerative forms of OH, such as diabetes mellitus, lupus, autoimmune aetiology, or amyloidosis; (4) incomplete data on the primary study outcome; and (5) refusal to participate in the study. This study was approved by the Ethics Committee of PUMCH (ethics number: JS-3287& K3279). All participants followed the voluntary principle and signed an informed consent. In this study, PD patients who participated in MDT (Multi-disciplinary Treatment) consultation were not included because most of them were evaluated perioperatively or followed up postoperatively with Deep Brain Stimulation (DBS). The final sample comprised 86 participants.

OH was diagnosed via active lying-to-standing blood pressure measurement in the consulting room. On examination day, participants were instructed to avoid caffeine, alcohol, and nicotine intake on. Prior to measurement, patients emptied their bladder and bowels. Blood pressure and heart rate were recorded using an electronic sphygmomanometer (Omron™) after 5 min in the supine position, and 3 min after standing. OH was defined according to established criteria as a decrease in systolic BP ≥ 20 mmHg, or a decrease in diastolic BP ≥ 10 mmHg within 3 min of standing (Schatz et al., 1996). NOH was identified when the Δ HR/ Δ SBP ratio was ≤0.5 bpm/mmHg (Fanciulli et al., 2020). Patients were then classified into an OH group and a non-OH group based on these criteria.

### 2.2 Clinical assessment

Demographic and clinical data, including age, sex, disease duration, and body mass index (BMI), were collected via standardized interviews. Functional status was evaluated using the modified Barthel Index (simplified Chinese version), a tool that measures basic Activities of Daily Living (ADL). Scores were categorized as: 0–20 (very severe impairment), 21–45 (severe impairment), 46–70 (moderate impairment), 71–99 (mild impairment), and 100 (full independence) (Shinar et al., 1987).

Owing to the diverse underlying aetiologies among the atypical parkinsonian patients evaluated in the MDT clinic (which included MSA, PSP, and other APS), and to focus on functional impairment as well as comparability of scores, the severity of nonmotor and motor symptoms were assessed via the Movement Disorder Society-Unified Parkinson’s Disease Rating Scale (MDS-UPDRS) part I (nonmotor experiences of daily living, nM-EDL) and part III (motor experiences of daily living, m-EDL). For patients taking Parkinson’s drugs, the LED was calculated at the time of the examination.

All patients were assessed using a 10-question-OH screening questionnaire (attachments required) (Gibbons et al., 2017) to identify potential symptoms associated with OH.

#### 2.2.1 Body composition measurement

Body composition was assessed using by a bioelectrical impedance analyzer (BIA Inbody770). Measurements included total, upper and lower limb fat-free mass (FFM), skeletal muscle mass (SM), total water (TW), intracellular water (IW), and extracellular water content (EW). As height significantly influences muscle mass, indices were derived by dividing each parameter by height squared (m^2^), in accordance with the AWGS2019 guidelines for muscle mass evaluation (Chen et al., 2020) —for example, FFMI = FFM (kg)/height (m)^2^ (Allcock et al., 2004). Accordingly, the analyser parameters comprised the overall, upper limb, lower limbs, and trunk fat-free mass index (FFMI, FFMI-up, FFMI-low, FFMI-trunk), the overall skeletal muscle index (SMI) and appendicular skeletal muscle index (AMI), the total water index of the overall and by region (TWI, UL, TWI, LLTWI, and TTWI), and intracellular and the extracellular water indices overall and by region (TIWI, UlIWI, and LLIWI; TEWI, UEWI, and LLEWI). Body composition measurements were performed immediately following supine-standing blood pressure assessment.

#### 2.2.2 Muscle strength

A hydraulic hand dynamometer (Xiangshan™) was used to assess grip strength. Participants were seated with the tested upper limb perpendicular to the floor and instructed to squeeze the device with maximum force. This procedure was repeated twice for each hand, and the highest reading from each side was recorded in kilograms (kg).

#### 2.2.3 Assessment of sarcopenia

The risk of sarcopenia was screened using the five-item SARC-F questionnaire, a tool recommended by the European Working Group on Sarcopenia in Older People (EWGSOP) (Cruz-Jentoft et al., 2019). Each item is scored 0–2, yielding a total score of 0–10; a score of ≥4 indicates elevated risk of sarcopenia. Sarcopenia was further diagnosed according to the AWGS2019 criteria (Cruz-Jentoft et al., 2019): low grip strength (<28.0 kg for men, <18.0 kg for women) defined pre-sarcopenia, while the combination of low grip strength and a low Appendix skeletal muscle index (AMI < 7.0 kg/m^2^ for men, <5.7 kg/m^2^ for women) confirmed sarcopenia.

### 2.3 Main variables of interest and rationale

The primary variable of interest in this study was the appendicular skeletal muscle index (AMI). The selection of AMI was based on two main rationales. Clinically, AMI is a core and widely accepted diagnostic criterion for sarcopenia, making it a highly standardized metric for evaluating muscle loss. Physiologically, skeletal muscle mass represents a critical biological infrastructure that acts as a physical peripheral pump to facilitate venous return upon standing and serves as the largest reservoir for intracellular water to buffer systemic blood volume fluctuations. Secondary variables of interest included other comprehensive body composition metrics: fat-free mass index (FFMI), skeletal muscle index (SMI), upper limb fat-free mass index (FFMI-up), lower limb fat-free mass index (FFMILOW), and trunk fat-free mass index (TFMI-trunk).

### 2.4 Statistical analysis

Continuous variables with normal distribution are presented as means ± standard deviations and compared using the independent-sample *t*-test. Skewed data are expressed as median (interquartile ranges) and analysed with the Mann–Whitney U test. Categorical data are presented as *n* (%), and compared using the chi-square test or Kruskal–Wall is test, as appropriate. Pearson’s correlation analysis was conducted to evaluate correlation among variables, visualized using a heatmap. To identify the most relevant risk factors for OH diagnosis, variable selection was performed using elastic-net regression, which combines Lasso and Ridge regularization techniques. This approach is particularly suited for settings with a high number of predictors relative to sample size or in the presence of multicollinearity. The selected features were then ranked by importance using support-vector machine (SVM) model, with performance validated via 5-fold cross-validation. All analyses were conducted in SPSS 25.0, with a significance level set at *α* = 0.05. A *p*-value < 0.05 was considered statistically significant.

## 3 Results

### 3.1 Results of the clinical evaluation

Among a total of 86 participants with neurodegenerative diseases (Table 1), there were 50 patients with MSA (25 cases each for types P and C), 15 with PSP, and the rest with APS (*n* = 21, including 2 patients with DLB and 19 patients with undifferentiated atypical parkinsonism). Nearly half of the participants (*n* = 39, 45.3%) were identified with a clinical diagnosis of OH. In terms of neurodegenerative disease classification, significant difference of OH between these groups was observed (H = 9.029, *p* = 0.007), MSA patients represented the greatest proportion (*n* = 29, 58.0%), followed by APS group (*n* = 8, 38.1%) and PSP group (*n* = 2, 13.3%).

**Table 1:** Baseline clinical information for the OH and non-OH groups.

Variables	Non-OH, *n* (%) (*N* = 47)	OH, *n* (%) (*N* = 39)	Total, *n* (%) (*N* = 86)	*p* value
MSA	21 (42)	29 (58)	50	0.007*
MSA-P	9 (36)	16 (64)	25	0.007*
MSA-C	12 (48)	13 (52)	25	0.007*
PSP	13 (86.7)	2 (13.3)	15	0.007*
APS	13 (61.9)	8 (38.1)	21	0.007*
OH screening	3 (1, 5)	4 (3, 6)	4 (2, 5)	0.011*
Gender (F/M)	18/29	21/18	39/47	0.221
Age	63.96 ± 7.47	59.74 ± 10.34	62.05 ± 9.08	0.037*
Disease duration (yr)	2.96 ± 1.35	2.61 ± 1.66	2.80 ± 1.50	0.285
LEDD	335.03 ± 290.06	312.39 ± 345.92	324.76 ± 314.89	0.742
BMI	24.99 ± 3.72	24.44 ± 3.82	24.74 ± 3.76	0.503
ADL	90 (62.5, 95)	70 (57.5, 87.5)	77.5 (60.95)	0.081
m-EDL	20.43 ± 10.75	22.38 ± 10.74	21.31 ± 10.72	0.402
nm-EDL	9.13 ± 4.43	9.31 ± 4.58	9.21 ± 4.47	0.854

ADL, modified Barthel index; APS, atypical Parkinsonian syndrome, (other atypical Parkinsonian syndromes); m-EDL: motor experiences of daily living on the MDS-UPDRS, MSA, multiple system atrophy; MSA-C, MSA with cerebellar-type; MSA-P, MSA with parkinsonian-type; nM-EDL: nonmotor experiences of daily living on the MDS-UPDRS; PSP, progressive supranuclear palsy; LEDD, levodopa equivalent daily dose. OH screening: refers to the total score of a 10-item orthostatic hypotension screening questionnaire (Gibbons et al., 2017), where each affirmative response equals 1 point (maximum score of 10). Data are presented as median (interquartile range).

There was no significant difference between OH and non-OH groups in terms of the sex ratio, disease duration, LED, ADL, exercise score or non-exercise score. However, the OH patients were significantly younger (59.74 ± 10.34 vs. 63.96 ± 7.47, *p* = 0.037).

### 3.2 Results of the body composition assessment

By comparing the muscle and body compositions of OH and non-OH patients, there were significant differences in the primary and secondary indicators of SMI, FFMIUP and FFMILOW. Moreover, we found significant differences in the intracellular and extracellular water indices of some limbs (Table 2). The appendicular skeletal muscle index and total water, intracellular water and extracellular water indices of OH patients were significantly lower than those of non-OH patients, but there was no statistical difference in terms of grip strength or the oedema index between the two groups. Among the 79 patients who completed the SARC-F questionaire, 66 were identified as being at risk for sarcopenia. Of those, 35 were normotensive and 31 had OH; however, no statistically significant difference was observed between the two groups (*p* = 0.282). According to the AWGS2019 diagnostic criteria, 9 patients (11.7%) were diagnosed with sarcopenia (6 patients in the OH group and 3 patients in the non-OH group, *p* = 0.124).

**Table 2:** Results of muscle assessment in the OH and non-OH groups.

No	Variables	Implications	Total (*N* = 86)	Non-OH (*N* = 47)	OH (*N* = 39)	*p* value
1	SARC-F	SARC-F score	6 (4, 7.75)	6 (4, 7)	6 (5, 8)	0.282
	SARC-*F* ≥ 4		66 (79)	35 (44)	31 (35)	0.282
2	OH screening	OH score	3 (1, 5)	4 (3, 6)	4 (2, 5)	0.011
3	Strength	Grip strength	22.4 (16.92, 28.97)	22.7 (18.25, 29.7)	20.9 (16.05, 26.85)	0.302
	Sarcopenia AWGS2019		9 (77)	3 (44)	6 (33)	0.124
4	FFMI	Fat-Free Mass/Height^2^	17 ± 2.13	17.41 ± 2.12	16.5 ± 2.05	0.045*
5	FFMIUP	Fat Free Mass of Upper Limbs /Height^2^	1.81 ± 0.37	1.89 ± 0.37	1.73 ± 0.37	0.048
6	FFMITRUNK	Fat Free Mass of Trunk/Height^2^	7.66 ± 0.99	7.85 ± 0.97	7.44 ± 0.98	0.058
7	FFMILOW	Fat-Free Mass of Lower Limbs /Height^2^	5.27 ± 0.78	5.42 ± 0.77	5.09 ± 0.75	0.050
8	SMI	Skeletal Muscle/Height^2^	9.16 ± 1.31	9.41 ± 1.29	8.85 ± 1.27	0.045*
9	AMI	Fat Free Mass of Appendicular Limbs /Height^2^	7.09 ± 1.1	7.31 ± 1.09	6.82 ± 1.06	0.040*
10	TWI	Total Water/Height^2^	12.54 ± 1.58	12.86 ± 1.58	12.17 ± 1.52	0.042*
11	ULTWI	Total Water of Upper Limbs/Height^2^	1.41 ± 0.29	1.47 ± 0.29	1.35 ± 0.29	0.048*
12	TTWI	Total Water of Trunk/Height^2^	6.00 ± 0.77	6.15 ± 0.76	5.83 ± 0.77	0.056
13	LLTWI	Total Water of Lower Limbs /Height^2^	4.13 ± 0.61	4.25 ± 0.60	3.99 ± 0.59	0.048*
14	ULIWI	Intracellular Water of Upper Limbs/Height^2^	0.87 ± 0.18	0.91 ± 0.18	0.83 ± 0.18	0.043*
15	TIWI	Intracellular Water of Trunk /Height^2^	3.63 ± 0.49	3.72 ± 0.48	3.52 ± 0.48	0.062
16	LLIWI	Intracellular Water of Lower Limbs/Height^2^	2.49 ± 0.37	2.56 ± 0.37	2.4 ± 0.36	0.053
17	ULEWI	Extracellular Water of Upper Limbs/Height^2^	0.54 ± 0.11	0.56 ± 0.11	0.52 ± 0.11	0.058
18	TEWI	Extracellular Water of Trunk/Height^2^	2.37 ± 0.29	2.43 ± 0.29	2.31 ± 0.29	0.052
19	LLEWI	Extracellular Water of Lower Limbs/Height^2^	1.65 ± 0.24	1.69 ± 0.24	1.59 ± 0.23	0.045*
20	ECW/TCW	Oedema Index	0.395 ± 0.007	0.395 ± 0.006	0.395 ± 0.008	0.726

AMI, appendicular skeletal muscle index; AWGS2019, Asian Working Group for Sarcopenia 2019; ECW/TCW, extracellular water to total body water ratio; FFMI, fat-free mass index; FFMILOW, lower limb fat-free mass index; FFMITRUNK, trunk fat-free mass index; FFMIUP, upper limb fat-free mass index; LLEWI, lower limb extracellular water index; LLIWI, lower limb intracellular water index; LLTWI, lower limb total water index; OH, orthostatic hypotension; SARC-F, Strength, Assistance with walking, Rise from a chair, Climb stairs and Falls questionnaire; SMI, skeletal muscle index; TEWI, trunk extracellular water index; TIWI, trunk intracellular water index; TTWI, trunk total water index; TWI, total water index; ULEWI, upper limb extracellular water index; ULIWI, upper limb intracellular water index; ULTWI, upper limb total water index.

### 3.3 Body composition: OH-related risk factors and ranking of importance

**Figure 1:**
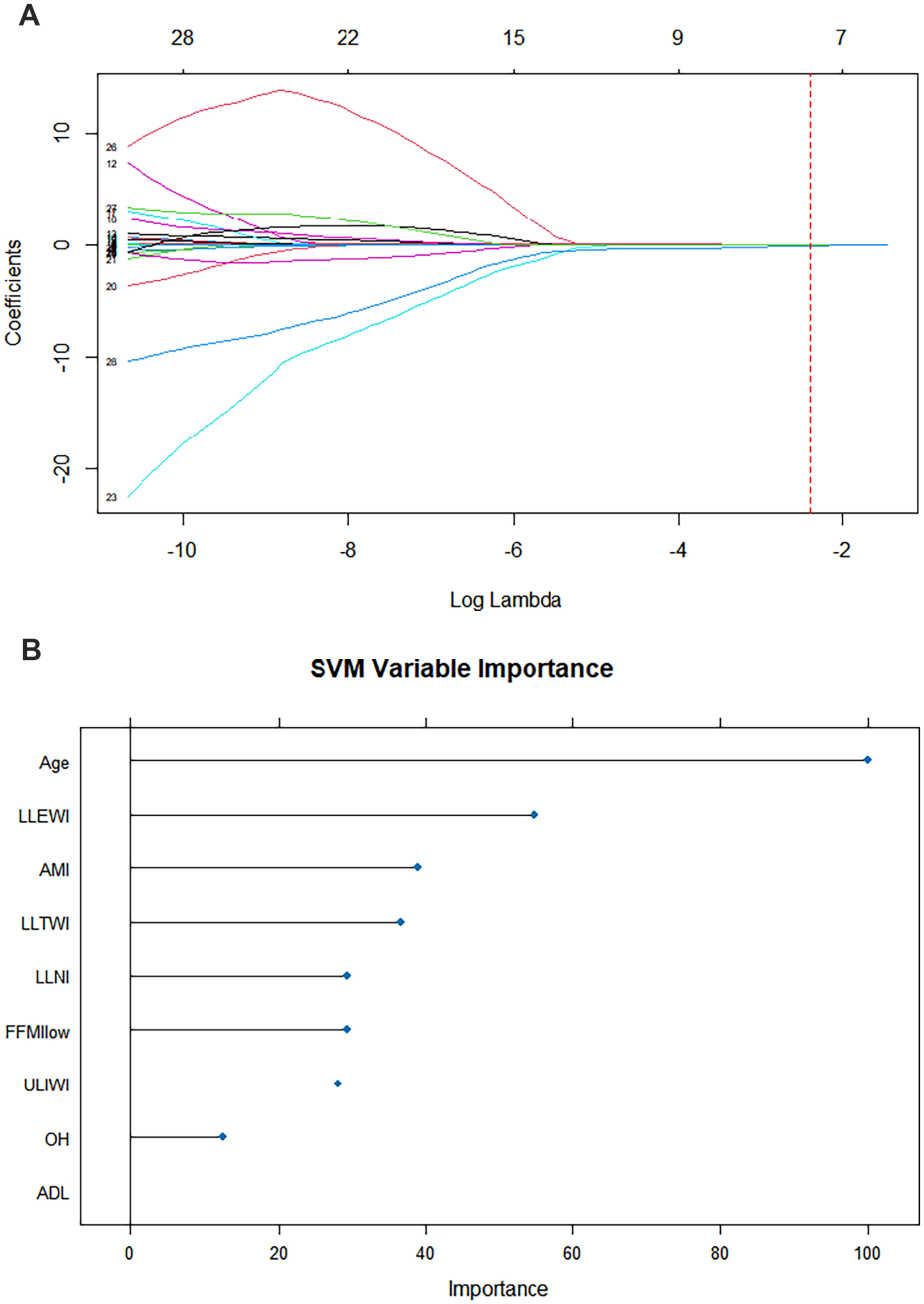
Variable selection and feature importance ranking for predicting Orthostatic Hypotension (OH). **(A)** Elastic Network Variable Screening: The coefficient profiles of the clinical and body composition variables are plotted against the Log Lambda sequence. Each colored line represents a distinct variable. The vertical red dashed line indicates the optimal lambda value chosen by cross-validation, which minimizes the mean squared error and successfully identifies the most robust predictors (non-zero coefficients) while shrinking irrelevant variables to zero. **(B)** SVM Feature Importance: The variables selected from the Elastic Net model **(A)** were ranked according to their predictive importance using a Support Vector Machine (SVM) model. The *x*-axis represents the relative variable importance score (scaled to a maximum of 100). Age, Lower Limb Extracellular Water Index (LLEWI), and Appendicular Skeletal Muscle Index (AMI) were identified as the top three most important predictors for OH.

**Figure 2:**
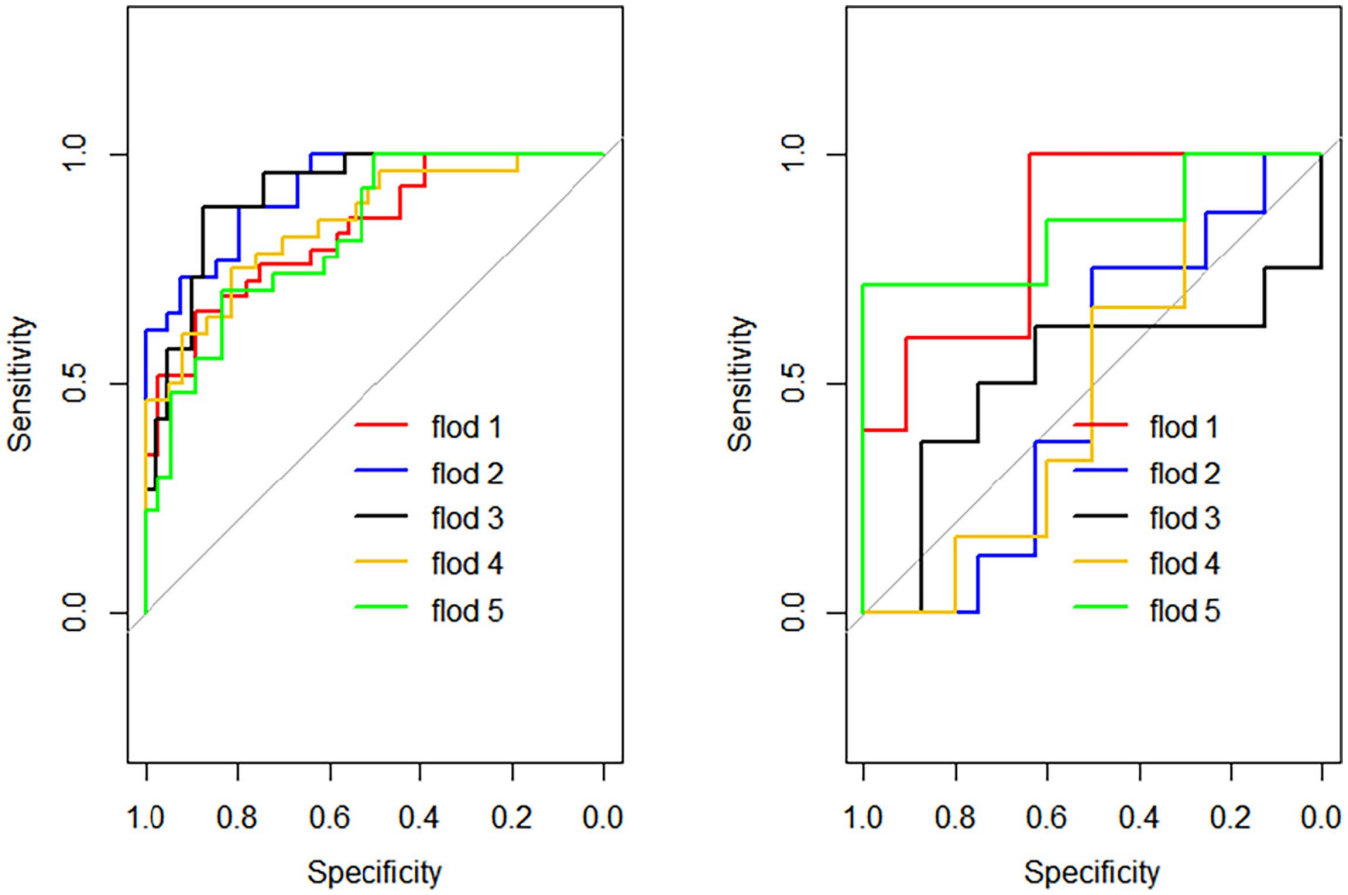
SVM predictive assessment via ROC curve analysis. The figure displays the Receiver Operating Characteristic (ROC) curves generated during the 5-fold cross-validation process to evaluate the performance of the Support Vector Machine (SVM) model. The left panel illustrates the ROC curves of the training sets across the five distinct folds, while the right panel shows the corresponding ROC curves of the validation sets. The *x*-axis represents 1-Specificity (False Positive Rate), and the y-axis represents Sensitivity (True Positive Rate). The different colored lines (folds 1 to 5) represent each individual data split during the cross-validation. The consistency and trajectory of the Area Under the Curve (AUC) across the folds demonstrate the high predictive stability and robustness of the body composition-based model in identifying orthostatic hypotension (OH).

A correlation analysis of all 86 patients was conducted. Age, disease course, height and weight were used as control variables to determine a significant correlation between each index existed. The relevant variables screened by the elastic network were age, ADL, OH, FFMILOW, AMI, lower limb total water index (LLTWI), upper limb intracellular water index (ULIWI), and lower limb extracellular water index (LLEWI) (Figure 1A). The variables obtained after the elastic network screening were ranked according to their importance via the SVM method, with age being the most important variable, followed by LLEWI, AMI, LLTWI, FFMILOW, ULIWI, OH, and ADL (Figure 1B). Fivefold cross-validation was further applied to verify the predictive validity of this ranking (Table 3; Figure 2).

**Table 3:** SVM 5-fold cross-validation results.

K-fold	Training set					Validation set				
	Events	Sample size	AUC	Low	Up	Events	Sample size	AUC	Low	Up
Fold 1	31	70	0.835	0.738	0.931	8	16	0.836	0.622	1.000
Fold 2	29	70	0.923	0.863	0.983	10	16	0.484	0.167	0.801
Fold 3	34	70	0.916	0.850	0.982	5	16	0.515	0.192	0.840
Fold 4	30	70	0.850	0.756	0.944	9	16	0.500	0.200	0.799
Fold 5	31	63	0.825	0.725	0.925	7	22	0.842	0.620	1.000

AUC, Area Under the Receiver Operating Characteristic curve; Low / Up, the lower and upper bounds of the 95% confidence interval (CI) for the AUC; Events, the number of patients with orthostatic hypotension (OH); Sample size, the number of cases in each cross-validation fold. This table demonstrates the predictive stability and performance of the Support Vector Machine (SVM) model across both training and validation sets during the 5-fold cross-validation process.

### 3.4 Risk factors associated with 3-minute supine-standing systolic blood pressure differences

To explore the effects of muscle-related factors on systolic blood pressure, we performed stepwise linear regression with 3-min systolic blood pressure as the dependent variable, and AMI and OH screening scores as the independent variables in all 86 patients. The model yielded *f* = 6.80, *p* = 0.02, indicating that the FFMI and OH scores influence the difference in systolic blood pressure. After adjustment for R2.0.133, i.e., this model explained 13.3% of the difference in systolic blood pressure with Ami (Expect (b) = −4.69, *p* = 0.008) and the OH score (Expect (b) = 2.03, *p* = 0.025). Diagnostic examination and residual analysis confirmed that, the model conformed to the assumption of linear regression, AMI and OH scores were significant predictors of the 3-min systolic blood pressure difference. Other factors, such as the skeletal muscle index, upper limb, lower limb, and total FFMI, were also revealed to serve as predictors of differences in systolic blood pressure. The remaining factors were not included in the prediction model because of their collinearity. Additional data are available in the Supplementary Table.

## 4 Discussion

This study investigated risk factors for OH from a body composition perspective in 86 patients with neurodegenerative diseases, constituting a large sample than previous reports (Bougea, 2023). To our best knowledge, this is the first large-scale study conducted in a parkinsonism-related multidisciplinary (PD-MDT) clinic that included non-Parkinson’s patients. The main findings were as follows: First, OH was common in this cohort (45.3%), being most prevalent in patients with MSA (58%), but also observed in those with PSP (13.3%) and other atypical parkinsonian patients (38.1%). Second, only 15.4% of OH-positive patients met the diagnostic criteria for sarcopenia, indicating a disproportion between OH prevalence and overt sarcopenia. Third, indices of FFM, skeletal muscle, total water, and intra- and extracellular water were significantly correlated with OH, suggesting that body composition alteration precede a formal diagnosis of sarcopenia. Furthermore, elastic-net regression was used to screen body-composition and muscle-related parameters, with importance ranking performed via SVM. LLEWI ranked highest predictors. Linear regression confirmed that both AMI and OH status were significant predictors of the 3-min systolic blood pressure (SBP) difference upon standing.

### 4.1 OH and muscle

Although sarcopenia has been proposed as a risk factor for OH (Keskin et al., 2021; Zhang et al., 2022), our study did not find the significant difference between OH and non-OH groups. Instead, we observed a significant lower level of skeletal muscle and body water indices in OH patients. Regression analysis further revealed that a higher AMI was associated with a smaller orthostatic drop in systolic BP. Prior studies on muscle and OH have shown consistent association. A 2020 cross-sectional analysis of 511 older adults found significant differences in 1-to-5 min supine-to-standing systolic blood pressure changes of categories of robust, probable sarcopenia, and sarcopenia (Soysal et al., 2020). Similarly, Benton et al. (2021) reported a correlation between orthostatic blood pressure drop and muscle volume in 69 community-dwelling older individuals. Older adults with sarcopenia defined by low muscle strength and poor physical performance also demonstrated impaired blood pressure recovery after posture change compared to those with normal muscle group (Duggan et al., 2023a). Crucially, our study extends this relationship—previously observed primarily in community-dwelling older adults—to patients with neurodegenerative disorders, enhancing its generalizability.

Regarding the pathophysiological mechanism of muscle influence on orthostatic blood pressure, previous studies in community-dwelling older individuals have proposed that low muscle mass may contribute to OH (Benton et al., 2021). Standing up causes an initial increase in venous return through the effects of contraction of leg and abdominal muscles. The consequent sudden increase in right atrial pressure may contribute to the fall in systemic vascular resistance through a reflex effect (Wieling et al., 2007). Subsequent contraction of lower limb muscles enhances venous via the skeletal muscular pump, thereby helping to restore blood pressure and attenuate systolic blood pressure fluctuation (Sheikh et al., 2022a; Tanaka et al., 1996). Calf circumference—a surrogate for lower-extremity muscle mass—has been linked to skeletal muscle pump efficacy and calf blood flow (Stewart et al., 2004). Furthermore, manoeuvres that passively compress or activate lower-limb muscles have been shown to improve postural blood pressure changes in healthy women (Sheikh et al., 2022b). Recent literature emphasizes the importance of distinguishing between neurogenic and non-neurogenic contributions to OH. For instance, Campese et al. (2021) demonstrated that non-neurogenic morphometric factors (such as height) can critically influence the magnitude of orthostatic blood pressure drops independently of the severity of underlying autonomic failure. Our findings support a similar dual-hit mechanistic framework. In neurodegenerative disorders, central or peripheral autonomic impairment often blunt sympathetic reflex increasing cardiac output during standing. Against this backdrop of compromised neurogenic regulation, non-neurogenic peripheral factors—specifically skeletal muscle mass—become critical. Consequently, the role of muscle pump in supporting venous return and blood pressure stabilization may become even more vital. In this study, we observed notable differences in muscle volume indices, between OH and non-OH groups. Correlation analysis and linear regression models also confirmed that AMI significantly influenced the magnitude of systolic blood pressure drop upon standing. These findings suggest that muscle volume may serve as important compensatory reserves for neurodegenerative disease patients’ maintaining hemodynamic stability during postural change. This highlights the potential clinical relevance of monitoring and preserving muscle volume in this vulnerable population.

### 4.2 OH and segmental body water distribution

The current study discovered significantly lower LLEWI, LLTWI, ULIWI, TWI in OH patients. Notably, these segmental body water distribution data as related risk factors were ranked by importance using SVM model, LLEWI and LLTWI emerged as important contributor in the diagnostic model, whereas trunk water indices showed no significant association. A possible explanation is that patients with neurodegenerative related OH may exhibit reduce total body water (Dmitrieva et al., 2024). In the setting of autonomic dysfunction, fluid retention represents an adaptive response to diminished cardiac output (Yu et al., 2024; Cihoric et al., 2023). To preserve cardiac filling and output, body water may redistribute centripetally, resulting in a reduction of limb water content, without a marked change in trunk water volume. Consequently, the reduction in both total and extracellular water content in the lower limbs, coupled with muscle atrophy, impairs the capacity to counteract the abrupt decline in systemic vascular resistance. Furthermore, the depletion of extracellular fluid—comprising interstitial fluid and circulating blood—further diminishes venous return, thereby exacerbating orthostatic hypotension in neurodegenerative disorders. This observation suggests that monitoring the lower limb water indices in OH patients serve as a practical indicator of insufficient system hydration, warranting increased clinical attention.

### 4.3 OH in neurodegenerative disorders patients

Neurodegenerative diseases are frequently associated with varying degrees of autonomic dysfunction (Wang et al., 2025). The epidemiological profile and diagnostic significance of OH vary considerably across these disorders. A recent systematic review and meta-analysis by Moss Lopes et al. (2025) demonstrated that cardiovascular autonomic failure in *α*-synucleinopathies is frequently accompanied by a high prevalence of both OH and supine hypertension, reflecting a profound disruption of central and peripheral autonomic networks. Consistent with these established pathophysiological distinctions, the highest prevalence of OH is generally reported in MSA—a prominent α-synucleinopathy characterized by widespread, early autonomic degeneration- reaching approximately 75% in some studies and often serving as a core diagnostic feature (Xie et al., 2022). Conversely, findings for PSP have been less consistent. Reported OH rates in PSP range from 9% to 45% in pathologically confirmed PSP cohorts (Baschieri et al., 2023; Oliveira et al., 2019; Wenning et al., 1999), with objective detection rates varying widely (0%–45%) depending on assessment methods (Baschieri et al., 2023). In this study, subgroup analysis of OH prevalence yielded results consistent with prior observations: OH was present in 58% of MSA patients, but only 13.3% of PSP patients, and 38.1% of those with other APS. These findings reinforce that while autonomic failure is significantly more severe and prevalent in α-synucleinopathies, OH remains a clinically relevant and not uncommon manifestation in tauopathies and other neurodegenerative conditions beyond MSA. Given the multifactorial nature of OH and the absence of disease-modifying therapies, identifying modifiable contributing factors—particularly body composition—holds significant clinical value for optimizing patient management.

## 5 Limitations

This study has several limitations. First, while an association between muscle-related indices and orthostatic blood pressure changes was observed, the cross-sectional design precludes causal inference. Whether interventions aimed at improving muscle mass or composition can durably ameliorate lying-to-standing blood pressure differences and OH symptoms require confirmation through longitudinal or interventional studies. Second, the participants were recruited from a movement disorder multidisciplinary clinic, representing a clinically complex cohort that may not fully reflect the broader population of patients with parkinsonism. Third, our predictive models are exploratory, and BIA-derived indices can be transiently influenced by acute hydration and clinical factors, limiting their immediate clinical applicability. Fourth, the cohort’s neuropathological heterogeneity and varying prevalences of autonomic dysfunction precluded robust subgroup multivariate analyses. Finally, given the modest overall sample size—despite the rarity of MSA and PSP—our findings require validation in larger, multicentre cohorts.

## 6 Conclusion

In this observational study, we found that OH is not uncommon in patients with neurodegenerative diseases, including MSA and PSP. Patients with OH exhibited significantly lower FFMI, SMI and AMI compared with those without OH. Among body composition indices, lower-limb water indices and AMI were particularly associated with OH. While these findings suggest that a reduction in lower-limb water content could serve as an early indicator of OH risk, the exploratory nature of our analyses and BIA’s sensitivity to transient hydration shifts dictate that further longitudinal studies are required to confirm their clinical applicability.

## Data Availability

The original contributions presented in the study are included in the article/Supplementary material, further inquiries can be directed to the corresponding authors.
