# Supplementary material for: The association between body composition and orthostatic hypotension in patients with neurodegenerative disorders in parkinsonism-related multidisciplinary clinic
Source: Front Aging Neurosci. 2026 Jul 7;18:1830578. doi: 10.3389/fnagi.2026.1830578 (PMC13385498; doi:10.3389/fnagi.2026.1830578)
Supplement: Supplementary file 1 [file Data_Sheet_1.zip › Ethics Approval-1.pdf]

中国医学科学院北京协和医院伦理审查委员会

会议审查批件

伦理审查批件编号：JS-3287

项目名称	神经源性体位性低血压的病理生理机制队列研究		
项目来源	国家自然科学基金		
项目科室	神经科	项目负责人	王含
会议地点	教学楼 314 会议室	会议日期	2021-12-28

主要研究者资格评价：

可

研究方案评价：

合格

版本号：V1.0　　　　版本日期：2021-12-28

知情同意书评价：

合格

版本号：V1.0　　　　版本日期：2021-12-28.

受试者补偿措施评价：

可

其他：

NA.

出席人数 21 人，　弃权人数 0 人　　回避人数 0 人

投票结果			审查意见
	批准	21 票	☒ 批准
	修改后批准	0 票	☐ 修改后批准
	修改后再审	0 票	☐ 修改后再审
	不批准	0 票	☐ 不批准
	暂停或终止研究	0 票	☐ 暂停或终止研究

跟踪审查频率为：☐3 个月　☐ 6 个月　☒ 1 年　☐ 无　☐其他

记录人签字：[illegible]	主任委员/副主任委员签字：[illegible]

中国医学科学院北京协和医院伦理审查委员会

（印章：中国医学科学院北京协和医院 伦理审查委员会）

扫描全能王 创建

中国医学科学院北京协和医院伦理审查委员会

会议审查批件

伦理审查批件编号：JS-3287

审查附件页：	审批文件	版本号	版本日期
1. 临床研究方案	V1.0	2021-12-28.	
2. 受试者知情同意书	V1.0	2021-12-28.	

1. 本伦理委员会是独立的，并遵守《涉及人的生物医学研究伦理审查办法》（原国家卫生计生委令第11 号）及当地相关法规。所有出席的委员均在有效任职期间。
2. 本伦理委员会将对所审阅的临床研究资料和相关的内容保密，且与本研究项目无利益冲突。
3. 如果研究中发生任何严重不良事件请于24 小时内通知本伦理委员会。
4. 批件有效期为 1 年，请在有效期内启动研究并按相应跟踪审查频率及时上报临床研究进展情况。
5. 本伦理委员会地址：北京市东城区帅府园 1 号。联系人：徐辉。联系电话：010-69156874。

中国医学科学院北京协和医院伦理审查委员会

扫描全能王 创建
