# Supplementary material for: The association between body composition and orthostatic hypotension in patients with neurodegenerative disorders in parkinsonism-related multidisciplinary clinic
Source: Front Aging Neurosci. 2026 Jul 7;18:1830578. doi: 10.3389/fnagi.2026.1830578 (PMC13385498; doi:10.3389/fnagi.2026.1830578)
Supplement: Supplementary file 1 [file Data_Sheet_1.zip › Ethics Approval-2.pdf]

伦理批件号：I-23PJ1013

中国医学科学院北京协和医院伦理审查委员会批件

项目编号	K3279	受理号	K23C1918
医学研究题目	运动障碍病自主神经损害的病理生理机制队列研究		
题目简写	运动障碍病自主神经损害队列		
研究经费来源	校级/院级-北京协和医院中央高水平医院临床科研专项（专科提升）：运动障碍病诊断技术与治疗体系的优化与应用（2022-PUMCH-B-018）;		
项目科室	神经科	项目负责人	王含
研究类型	观察性研究	牵头单位	中国医学科学院北京协和医院
审查类别	复审	审查方式	快速审查
会议日期	NA	会议地点	NA

伦理审查决定：批准

批准日期：2023-06-29

跟踪审查频率：☐ 3个月　☐ 6个月　■ 1年　☐ 无　☐ 其他

说明：

1. *请按照GCP原则和伦理委员会批准的文件开展研究，保护受试者的健康与权益。*
2. *凡涉及人类遗传资源管理或者国家规定必须经有关部门审批的项目，均需在执行前向有关部门申报并获得批准。*
3. *研究过程中，对已批准的研究方案、知情同意书（如有）、招募材料（如有）等的任何修改以及研究者/研究项目负责人的变更，请提交修正案审查申请，均须得到伦理委员会审查批准后方可实施。*
4. *本中心发生的严重不良事件或严重影响受试者安全或权益的事件，请于研究者获知后的24小时内向伦理委员会作书面报告，伦理委员会有权对其评估做出新的决定。*
5. *本中心发生偏离方案时，请及时提交偏离方案报告。*
6. *本批件有效期1年，逾期未启动研究伦理批件失效。再次准备启动研究时，需重新提交伦理审查。*
7. *无论研究开始与否，请按照伦理委员会规定的跟踪审查频率，在截止日期前1个月提交跟踪审查报告。*
8. *暂停或提前终止研究，请及时提交暂停/终止研究报告。*
9. *完成临床研究，请提交结题报告。*

主任委员/副主任委员签字	[signature]	主任委员/副主任委员签字日期	2023-6-29

中国医学科学院北京协和医院伦理审查委员会

中国医学科学院北京协和医院伦理审查委员会批件

审查文件（注明版本号、版本日期）

1、伦理意见回复函《3. 科研伦理-复审申请表（伦理意见回复函）-2023-6-13.pdf》(1.0、2023-06-13)

2、知情同意书《科研-知情同意书-20230613.pdf》(1.0、2023-06-13)

声明：

1. 本伦理委员会的组成及工作程序符合《赫尔辛基宣言》、《人体生物医学研究国际道德指南》、《涉及人的生物医学研究伦理审查办法》、《药物临床试验质量管理规范》、《医疗器械临床试验质量管理规范》和《药物临床试验伦理审查工作指导原则》等国际伦理原则及我国相关规章和指导原则要求。
2. 所有参与审查的委员均在有效任职期间。
3. 所有参与审查的委员对所审阅的临床研究资料和相关的内容保密，且与本研究项目无利益冲突。
4. 本伦理委员会地址：北京东城区帅府园1号。联系人：李佳月/苗龙芳。联系电话：010-69156874/69155709。

中国医学科学院北京协和医院伦理审查委员会
