# Supplementary material for: The association between body composition and orthostatic hypotension in patients with neurodegenerative disorders in parkinsonism-related multidisciplinary clinic
Source: Front Aging Neurosci. 2026 Jul 7;18:1830578. doi: 10.3389/fnagi.2026.1830578 (PMC13385498; doi:10.3389/fnagi.2026.1830578)
Supplement: Supplementary file 1 [file Data_Sheet_1.zip › Machine Learning.docx]

**数据分析结果**

1. **基本信息**

两组患者在原发病, 年龄, OH, Strength, FFMI, FFMIup, FFMIlow, AMI, SMI, 总水指数, 上肢肌肉指数, 下肢肌肉指数, 上肢总水指数, 等存在显著差异(见表1)。

表1 纳入病例基本信息

| No | Variables | 变量 | Total  (n = 81) | Group0  (n = 47) | Group1  (n = 39) | p |
| --- | --- | --- | --- | --- | --- | --- |
| 1 | Disease | 原发病 |  |  |  | 0.007 |
|  | 1 |  | 50 (58) | 21 (45) | 29 (74) |  |
|  | 2 |  | 15 (17) | 13 (28) | 2 (5) |  |
|  | 3 |  | 21 (24) | 13 (28) | 8 (21) |  |
| 2 | Gender | 性别 |  |  |  | 0.221 |
|  | 0 |  | 39 (45) | 18 (38) | 21 (54) |  |
|  | 1 |  | 47 (55) | 29 (62) | 18 (46) |  |
| 3 | Age | 年龄 | 62.05 ± 9.08 | 63.96 ± 7.47 | 59.74 ± 10.34 | 0.037 |
| 4 | BMI | BMI | 24.74 ± 3.76 | 24.99 ± 3.72 | 24.44 ± 3.82 | 0.503 |
| 5 | ADL | ADL | 77.5 (60, 95) | 90 (62.5, 95) | 70 (57.5, 87.5) | 0.081 |
| 6 | M | M | 21.31 ± 10.72 | 20.43 ± 10.75 | 22.38 ± 10.74 | 0.402 |
| 7 | SARCF | SARCF | 6 (4, 7.75) | 6 (4, 7) | 6 (5, 8) | 0.282 |
| 8 | nM | nM | 9.21 ± 4.47 | 9.13 ± 4.43 | 9.31 ± 4.58 | 0.854 |
| 9 | OH | OH | 3 (1, 5) | 4 (3, 6) | 4 (2, 5) | 0.011 |
| 10 | Strength | 握力大 | 22.4 (16.92, 28.97) | 22.7 (18.25, 29.7) | 20.9 (16.05, 26.85) | 0.302 |
| 11 | FFMI | FFMI | 17 ± 2.13 | 17.41 ± 2.12 | 16.5 ± 2.05 | 0.045 |
| 12 | FFMItrunk | FFMItrunk | 7.66 ± 0.99 | 7.85 ± 0.97 | 7.44 ± 0.98 | 0.058 |
| 13 | FFMIup | FFMIup | 1.81 ± 0.37 | 1.89 ± 0.37 | 1.73 ± 0.37 | 0.048 |
| 14 | FFMIlow | FFMIlow | 5.27 ± 0.78 | 5.42 ± 0.77 | 5.09 ± 0.75 | 0.050 |
| 15 | AMI | AMI | 7.09 ± 1.1 | 7.31 ± 1.09 | 6.82 ± 1.06 | 0.040 |
| 16 | SMI | SMI | 9.16 ± 1.31 | 9.41 ± 1.29 | 8.85 ± 1.27 | 0.045 |
| 17 | TWI | 总水指数 | 12.54 ± 1.58 | 12.86 ± 1.58 | 12.17 ± 1.52 | 0.042 |
| 18 | ULMI | 上肢肌肉指数 | 1.81 ± 0.37 | 1.89 ± 0.37 | 1.73 ± 0.37 | 0.048 |
| 19 | LLNI | 下肢肌肉指数 | 5.27 ± 0.78 | 5.42 ± 0.77 | 5.09 ± 0.75 | 0.050 |
| 20 | TML | 躯干肌肉指数 | 7.66 ± 0.99 | 7.85 ± 0.97 | 7.44 ± 0.98 | 0.058 |
| 21 | ULTWI | 上肢总水指数 | 1.41 ± 0.29 | 1.47 ± 0.29 | 1.35 ± 0.29 | 0.048 |
| 22 | TTWI | 躯干总水指数 | 6.00 ± 0.77 | 6.15 ± 0.76 | 5.83 ± 0.77 | 0.056 |
| 23 | LLTWI | 下肢总水指数 | 4.13 ± 0.61 | 4.25 ± 0.60 | 3.99 ± 0.59 | 0.048 |
| 24 | ULIWI | 上肢细胞内水指数 | 0.87 ± 0.18 | 0.91 ± 0.18 | 0.83 ± 0.18 | 0.043 |
| 25 | TIWI | 躯干细胞内水指数 | 3.63 ± 0.49 | 3.72 ± 0.48 | 3.52 ± 0.48 | 0.062 |
| 26 | LLIWI | 下肢细胞内水指数 | 2.49 ± 0.37 | 2.56 ± 0.37 | 2.4 ± 0.36 | 0.053 |
| 27 | ULEWI | 上肢细胞外水指数 | 0.54 ± 0.11 | 0.56 ± 0.11 | 0.52 ± 0.11 | 0.058 |
| 28 | TEWI | 躯干细胞外水指数 | 2.37 ± 0.29 | 2.43 ± 0.29 | 2.31 ± 0.29 | 0.052 |
| 29 | LLEWI | 下肢细胞外水指数 | 1.65 ± 0.24 | 1.69 ± 0.24 | 1.59 ± 0.23 |  |

1. **相关性分析**

表中各指数间存在严重的相关性。

表2 各指数间的相关系数

|  | Strength | FFMI | FFMItrunk | FFMIup | FFMIlow | AMI | SMI | TWI | ULMI | LLNI | TML | ULTWI | TTWI | LLTWI | ULIWI | TIWI | LLIWI | ULEWI | TEWI | LLEWI |
| --- | --- | --- | --- | --- | --- | --- | --- | --- | --- | --- | --- | --- | --- | --- | --- | --- | --- | --- | --- | --- |
| Strength | 1.0 | 0.6 | 0.6 | 0.6 | 0.6 | 0.6 | 0.6 | 0.6 | 0.6 | 0.6 | 0.6 | 0.6 | 0.6 | 0.6 | 0.6 | 0.7 | 0.6 | 0.6 | 0.5 | 0.5 |
| FFMI | 0.6 | 1.0 | 0.9 | 0.9 | 0.9 | 1.0 | 1.0 | 1.0 | 0.9 | 0.9 | 0.9 | 0.9 | 0.9 | 0.9 | 0.9 | 0.9 | 0.9 | 0.9 | 0.9 | 0.9 |
| FFMItrunk | 0.6 | 0.9 | 1.0 | 1.0 | 0.8 | 0.9 | 0.9 | 0.9 | 1.0 | 0.8 | 1.0 | 1.0 | 1.0 | 0.8 | 1.0 | 1.0 | 0.8 | 1.0 | 1.0 | 0.7 |
| FFMIup | 0.6 | 0.9 | 1.0 | 1.0 | 0.8 | 0.9 | 1.0 | 0.9 | 1.0 | 0.8 | 1.0 | 1.0 | 1.0 | 0.8 | 1.0 | 1.0 | 0.8 | 1.0 | 1.0 | 0.8 |
| FFMIlow | 0.6 | 0.9 | 0.8 | 0.8 | 1.0 | 1.0 | 0.9 | 0.9 | 0.8 | 1.0 | 0.8 | 0.8 | 0.8 | 1.0 | 0.8 | 0.8 | 1.0 | 0.8 | 0.8 | 1.0 |
| AMI | 0.6 | 1.0 | 0.9 | 0.9 | 1.0 | 1.0 | 1.0 | 1.0 | 0.9 | 1.0 | 0.9 | 0.9 | 0.9 | 1.0 | 0.9 | 0.9 | 1.0 | 0.9 | 0.9 | 1.0 |
| SMI | 0.6 | 1.0 | 0.9 | 1.0 | 0.9 | 1.0 | 1.0 | 1.0 | 1.0 | 0.9 | 0.9 | 1.0 | 0.9 | 0.9 | 0.9 | 0.9 | 0.9 | 1.0 | 0.9 | 0.9 |
| TWI | 0.6 | 1.0 | 0.9 | 0.9 | 0.9 | 1.0 | 1.0 | 1.0 | 0.9 | 0.9 | 0.9 | 0.9 | 0.9 | 0.9 | 0.9 | 0.9 | 0.9 | 0.9 | 0.9 | 0.9 |
| ULMI | 0.6 | 0.9 | 1.0 | 1.0 | 0.8 | 0.9 | 1.0 | 0.9 | 1.0 | 0.8 | 1.0 | 1.0 | 1.0 | 0.8 | 1.0 | 1.0 | 0.8 | 1.0 | 1.0 | 0.8 |
| LLNI | 0.6 | 0.9 | 0.8 | 0.8 | 1.0 | 1.0 | 0.9 | 0.9 | 0.8 | 1.0 | 0.8 | 0.8 | 0.8 | 1.0 | 0.8 | 0.8 | 1.0 | 0.8 | 0.8 | 1.0 |
| TML | 0.6 | 0.9 | 1.0 | 1.0 | 0.8 | 0.9 | 0.9 | 0.9 | 1.0 | 0.8 | 1.0 | 1.0 | 1.0 | 0.8 | 1.0 | 1.0 | 0.8 | 1.0 | 1.0 | 0.7 |
| ULTWI | 0.6 | 0.9 | 1.0 | 1.0 | 0.8 | 0.9 | 1.0 | 0.9 | 1.0 | 0.8 | 1.0 | 1.0 | 1.0 | 0.8 | 1.0 | 1.0 | 0.8 | 1.0 | 1.0 | 0.8 |
| TTWI | 0.6 | 0.9 | 1.0 | 1.0 | 0.8 | 0.9 | 0.9 | 0.9 | 1.0 | 0.8 | 1.0 | 1.0 | 1.0 | 0.8 | 1.0 | 1.0 | 0.8 | 1.0 | 1.0 | 0.7 |
| LLTWI | 0.6 | 0.9 | 0.8 | 0.8 | 1.0 | 1.0 | 0.9 | 0.9 | 0.8 | 1.0 | 0.8 | 0.8 | 0.8 | 1.0 | 0.8 | 0.8 | 1.0 | 0.8 | 0.8 | 1.0 |
| ULIWI | 0.6 | 0.9 | 1.0 | 1.0 | 0.8 | 0.9 | 0.9 | 0.9 | 1.0 | 0.8 | 1.0 | 1.0 | 1.0 | 0.8 | 1.0 | 1.0 | 0.8 | 1.0 | 1.0 | 0.8 |
| TIWI | 0.7 | 0.9 | 1.0 | 1.0 | 0.8 | 0.9 | 0.9 | 0.9 | 1.0 | 0.8 | 1.0 | 1.0 | 1.0 | 0.8 | 1.0 | 1.0 | 0.8 | 1.0 | 1.0 | 0.7 |
| LLIWI | 0.6 | 0.9 | 0.8 | 0.8 | 1.0 | 1.0 | 0.9 | 0.9 | 0.8 | 1.0 | 0.8 | 0.8 | 0.8 | 1.0 | 0.8 | 0.8 | 1.0 | 0.8 | 0.8 | 1.0 |
| ULEWI | 0.6 | 0.9 | 1.0 | 1.0 | 0.8 | 0.9 | 1.0 | 0.9 | 1.0 | 0.8 | 1.0 | 1.0 | 1.0 | 0.8 | 1.0 | 1.0 | 0.8 | 1.0 | 1.0 | 0.8 |
| TEWI | 0.5 | 0.9 | 1.0 | 1.0 | 0.8 | 0.9 | 0.9 | 0.9 | 1.0 | 0.8 | 1.0 | 1.0 | 1.0 | 0.8 | 1.0 | 1.0 | 0.8 | 1.0 | 1.0 | 0.8 |
| LLEWI | 0.5 | 0.9 | 0.7 | 0.8 | 1.0 | 1.0 | 0.9 | 0.9 | 0.8 | 1.0 | 0.7 | 0.8 | 0.7 | 1.0 | 0.8 | 0.7 | 1.0 | 0.8 | 0.8 | 1.0 |


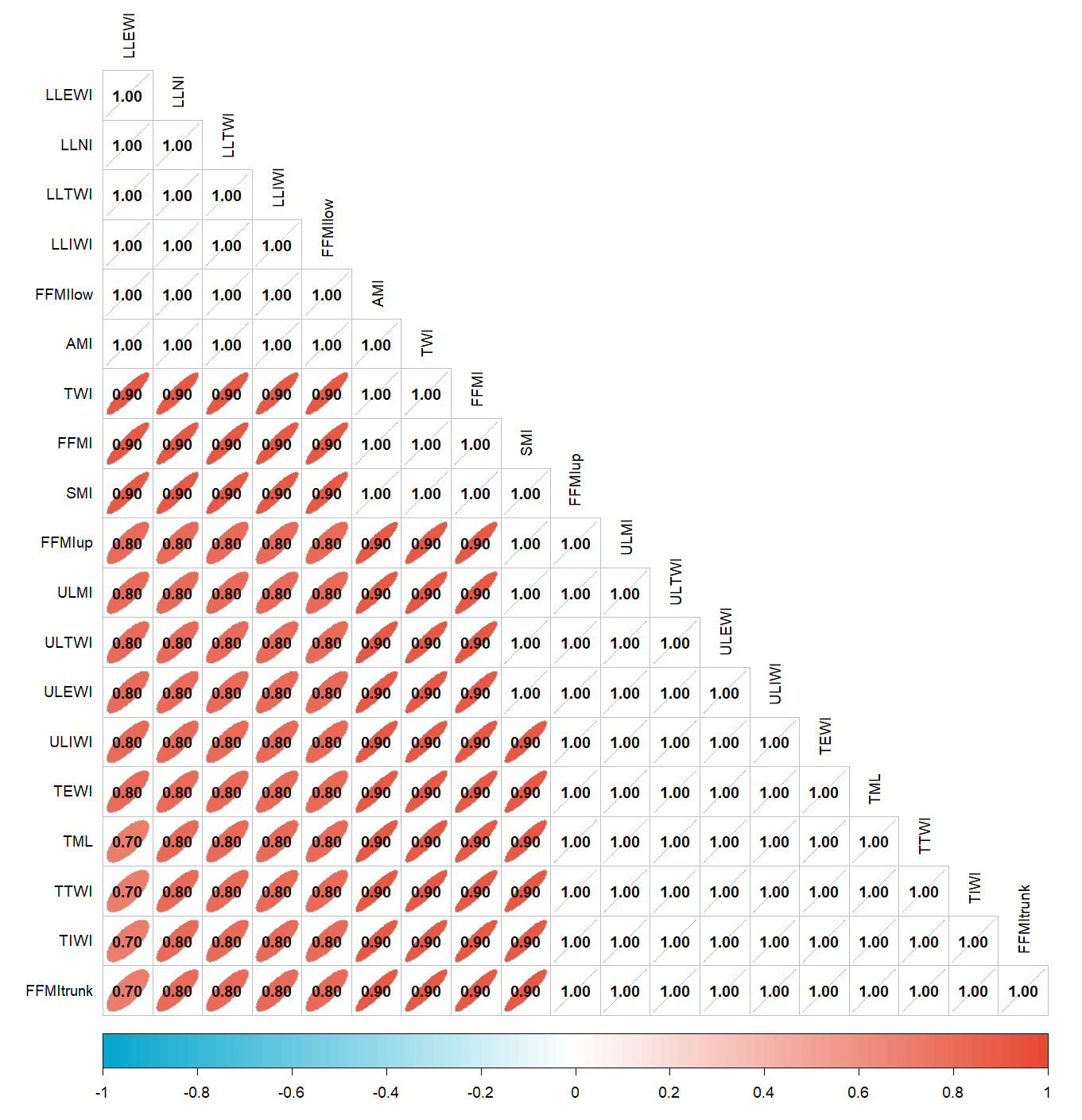


图1 各指数间的相关性分析

1. **弹性网络筛选变量**

我们选择弹性网络进行变量筛选，弹性网络是一种用于线性回归模型的正则化方法，它结合了Lasso和Ridge回归的优点。旨在解决当预测变量（特征）数量大于观测样本数量，或者多个特征之间存在高度相关时的建模问题。弹性网络筛选的相关变量为Age、ADL、OH、FFMIlow、AMI、LLNI(下肢肌肉指数)、LLTWI(下肢总水指数)、ULIWI(上肢细胞内水指数)、LLEWI(上肢细胞外水指数)。


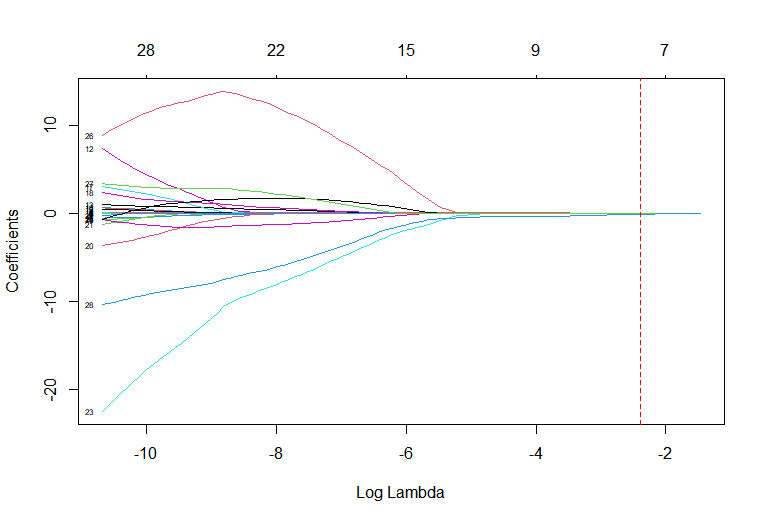


图2 弹性网络的变量筛选结果

1. **变量重要性排序**

本研究采用随机森林、支持向量机和XGBoost进行变量重要性排序。

随机森林按增加准确性的排序结果显示，变量重要性依次为Age、OH、ADL、LLEWI(上肢细胞外水指数)、LLTWI(下肢总水指数)、LLNI(下肢肌肉指数)、AMI、FFMIlow、ULIWI(上肢细胞内水指数)(图3)。

支持向量机排序结果显示，变量重要性依次为Age、LLEWI(上肢细胞外水指数)、AMI（）、LLTWI(下肢总水指数)、LLNI(下肢肌肉指数)、FFMIlow、ULIWI(上肢细胞内水指数)、OH、ADL(图4)。

XGBoost排序结果显示，变量重要性依次为Age、ADL、ULIWI(上肢细胞内水指数)、LLEWI(上肢细胞外水指数)、OH、LLTWI(下肢总水指数)、AMI、FFMIlow、LLNI(下肢肌肉指数)(图5)。

这三个模型重要性排序似乎存在一定的差异，然而，支持向量机的准确性似乎更好。


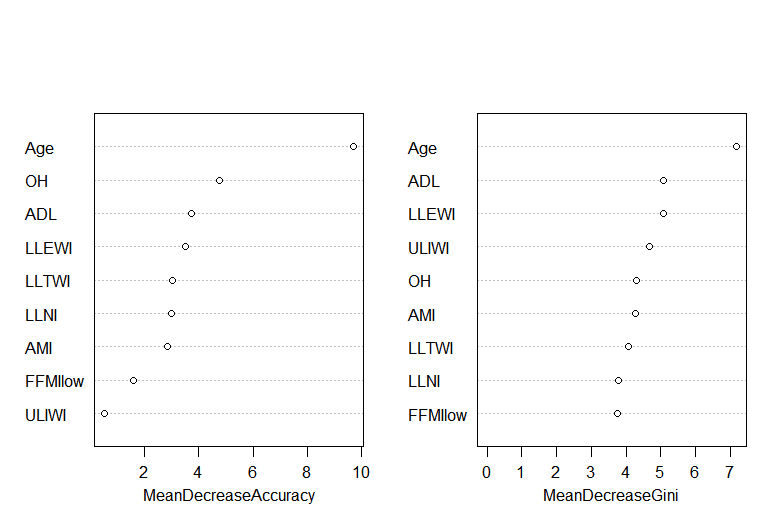


图3 随机森林变量重要性排序结果


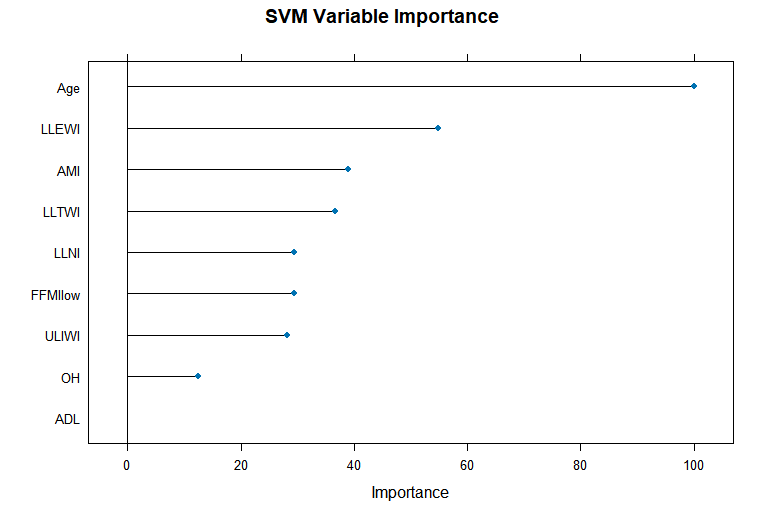


图4 支持向量机变量重要性排序结果


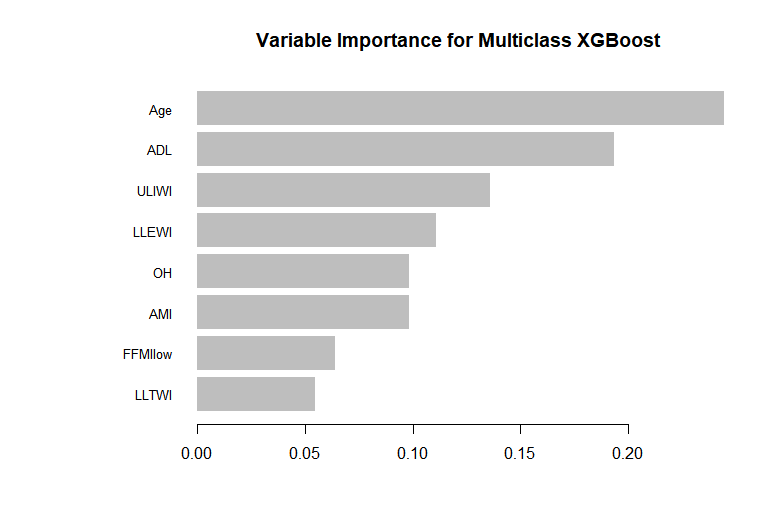


图5 XGBoost变量重要性排序结果

1. **机器学习的构建与k-折交叉验证**

表3 RF的5折交叉验证结果

| k-折 | 训练集 | | | | | 训练集 | | | | |
| --- | --- | --- | --- | --- | --- | --- | --- | --- | --- | --- |
|  | Events | samplesize | Auc | low | up | Events | samplesize | Auc | low | up |
| flod 1 | 31 | 70 | 0.523 | 0.370 | 0.676 | 8 | 16 | 0.763 | 0.515 | 1.000 |
| flod 2 | 29 | 70 | 0.639 | 0.485 | 0.793 | 10 | 16 | 0.554 | 0.247 | 0.863 |
| flod 3 | 34 | 70 | 0.684 | 0.550 | 0.817 | 5 | 16 | 0.484 | 0.160 | 0.808 |
| flod 4 | 30 | 70 | 0.610 | 0.462 | 0.757 | 9 | 16 | 0.550 | 0.229 | 0.871 |
| flod 5 | 31 | 63 | 0.521 | 0.367 | 0.675 | 7 | 22 | 0.771 | 0.521 | 1.00 |

表4 SVM的5折交叉验证结果

| k-折 | 训练集 | | | | | 验证集 | | | | |
| --- | --- | --- | --- | --- | --- | --- | --- | --- | --- | --- |
|  | Events | samplesize | Auc | low | up | Events | samplesize | Auc | low | up |
| flod 1 | 31 | 70 | 0.835 | 0.738 | 0.931 | 8 | 16 | 0.836 | 0.622 | 1.000 |
| flod 2 | 29 | 70 | 0.923 | 0.863 | 0.983 | 10 | 16 | 0.484 | 0.167 | 0.801 |
| flod 3 | 34 | 70 | 0.916 | 0.850 | 0.982 | 5 | 16 | 0.515 | 0.192 | 0.840 |
| flod 4 | 30 | 70 | 0.850 | 0.756 | 0.944 | 9 | 16 | 0.500 | 0.200 | 0.799 |
| flod 5 | 31 | 63 | 0.825 | 0.725 | 0.925 | 7 | 22 | 0.842 | 0.620 | 1.000 |

表5 XGBoost的5折交叉验证结果

| k-折 | 训练集 | | | | | 训练集 | | | | |
| --- | --- | --- | --- | --- | --- | --- | --- | --- | --- | --- |
|  | Events | samplesize | Auc | low | up | Events | samplesize | Auc | low | up |
| flod 1 | 31 | 70 | 0.913 | 0.845 | 0.981 | 8 | 16 | 0.515 | 0.198 | 0.832 |
| flod 2 | 29 | 70 | 0.914 | 0.849 | 0.978 | 10 | 16 | 0.517 | 0.154 | 0.878 |
| flod 3 | 34 | 70 | 0.890 | 0.812 | 0.968 | 5 | 16 | 0.429 | 0.113 | 0.743 |
| flod 4 | 30 | 70 | 0.909 | 0.834 | 0.985 | 9 | 16 | 0.603 | 0.277 | 0.929 |
| flod 5 | 31 | 63 | 0.894 | 0.808 | 0.980 | 7 | 22 | 0.773 | 0.515 | 1.000 |


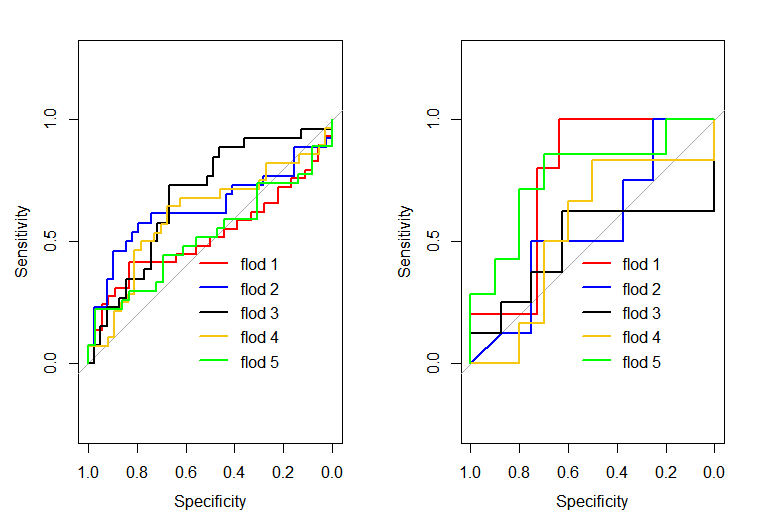


图6 RF的5折交叉验证的Roc曲线


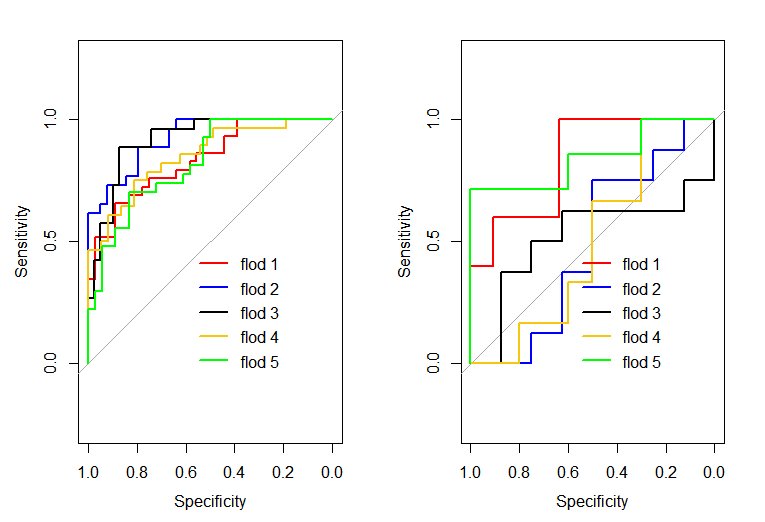


图7 SVM的5折交叉验证的Roc曲线


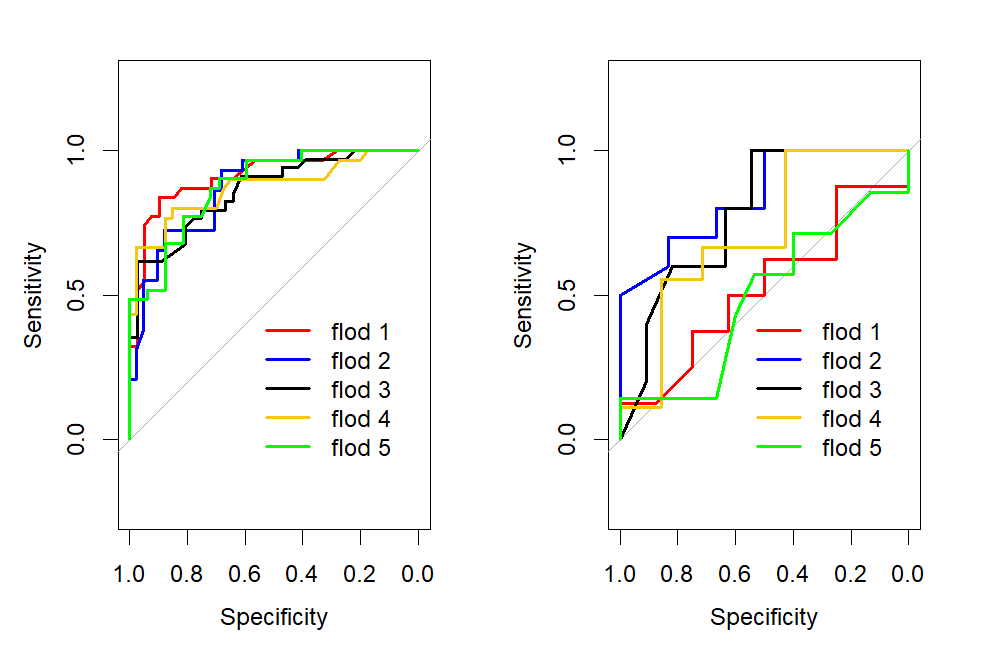


图8 XGBoost的5折交叉验证的Roc曲线
